# Double membrane platelet-rich fibrin (PRF) – Synovium succeeds in regenerating cartilage defect at the knee: An experimental study on rabbit

**DOI:** 10.1016/j.heliyon.2023.e13139

**Published:** 2023-01-22

**Authors:** Ahmad Taufik S, Bayu Tirta Dirja, Dwikora Novembri Utomo, Muhammad Andry Usman, Muhammad Sakti, Muhammad Ruksal Saleh, Mochammad Hatta, Budu

**Affiliations:** a Faculty of Medicine Mataram University, Mataram, Indonesia; b Department of Orthopaedic, Faculty of Medicine Airlangga University, Surabaya, Indonesia; c Department of Orthopaedic, Faculty of Medicine Hasanuddin University, Makasar, Indonesia; d Department of Molecular Biology and Immunology, Faculty of Medicine, Hasanuddin University, Makassar, Indonesia; e Department of Opthalmology, Faculty of Medicine, Hasanuddin University, Makassar, Indonesia

**Keywords:** Cartilage defect, Microfracture, Platelet-rich fibrin, Synovium

## Abstract

**Background:**

This study aims to prove the healing results (regeneration) in cartilage defects using a combination treatment of microfractures and transplantation synovium-platelet rich fibrin (S-PRF).

**Methods:**

A cartilage defect was made in the trochlear groove of the knee of adult New Zealand white rabbits, and was classified into three treatment groups. The group 1 was cartilage defect without treatment, 2 with microfracture treatment, and 3 with microfracture covered with a synovium-platelet rich fibrin (S-PRF) membrane. Twelve weeks after the intervention, the animals were macroscopically and histologically examined, and evaluated by the International Cartilage Repair Society (ICRS). Additionally, the expression of aggrecan and type 2 collagen was examined by real-time-PCR.

**Results:**

The ICSR scores for macroscopic were significantly higher in the microfracture and S-PRF transplant group than in the other groups. Also, the ICSR scores for histology were significantly higher in this group. The expression of aggrecan and type 2 collagen was higher in the group that received complete treatment.

**Conclusions:**

Microfractures and transplantation of synovium-platelet rich fibrin (S-PRF) can regenerate knee cartilage defects which have been shown to increase the expression of mRNA aggrecan and mRNA type 2 collagen resulting in excellent repair.

## 1 Introduction

Cartilage defects are serious problems in 60% of patients who complain of knee pain [1,2]. Healing or regeneration in joint cartilage is characterized by poor tissue because it has no vascularization, few cells, no basement membrane, and no nerve supply. Therefore, its nutrition only depends on the diffusion process [3]. The current treatment attempts to restore the normal cartilage structure involve using articular hyaline to completely fill the defect [4,5,6].

Various methods have been developed for the treatment of cartilage defects ranging from bone marrow stimulation techniques with microfracture repair to restoration techniques with auto or osteochondral allograft and autologous chondrocytes implantation (ACI) [7]. Currently, the most widely developed treatment method is tissue engineering-based therapy with mesenchymal stem cells (MSCs) grafting and matrix-induced autologous chondrocytes implantation (MACI) [4,8,9]. The use of ACI and MSC tissue engineering technology has several weaknesses, namely complex facilities to develop stem cells, high cost, as well as requires twice operation and time [8].

Microfracture is a bone marrow stimulation procedure in the treatment of joint cartilage defects in addition to subchondral drilling and abrasion techniques [10,11,12]. Studies have shown that this method provides healing with fibrocartilage tissue that is histologically and biomechanically below normal hyaline cartilage [13,14]. Therefore, various attempts have been made to improve the outcome of these microfractures, including adding (augmenting) natural biological substances, such as intra-articular MSC, platelet-rich plasma (PRP), platelet-rich fibrin (PRF), scaffold and others as adjuvants [9,15]. The combination of microfractures and biologics can promote the healing to hyaline-like cartilage [16].

Platelet-rich fibrin is a biomaterial product that is widely used in regenerative medicine. It contains a lot of platelets, growth factors, cytokines, and white blood cells (leukocytes). The growth factors include platelet-derived growth factor (PDGF), transforming growth factor-beta (TGF-β), insulin growth factor (IGF), and bone morphogenic protein (BMP). This growth factor has been shown to stimulate stem cells and differentiate into chondroblasts, osteoblasts, and other precursor cells [8,17]. PRF has recently been used more clinically than platelet-rich plasma due to its easier application form and more complete content. Furthermore, it contains protein-rich molecules as part of platelets and leukocytes which can reduce rejection reactions, has an antibacterial response, and accelerate tissue healing [17].

This study developed a method of treating joint cartilage defects with a combination of microfractures and synovium grafts as well as platelet-rich fibrin membrane (S-PRF). In this combination, synovium functions as a source of mesenchymal stem cells, while PRF membrane as the source of growth factors that regenerates (heal) cartilage defects. In vitro, these synovial cells have been shown to be chondrogenic and capable of differentiation into chondroblasts [18]. Microfractures aim to pave the way for progenitor cells, especially mesenchymal stem cells from the bone marrow to the cartilage defect site to accelerate healing. This combination of actions is expected to improve healing and the quality of the cartilage formed. Therefore, this study aims to prove the healing results (regeneration) in cartilage defects using a combination of microfracture treatment and synovium-platelet rich fibrin grafting.

## 2 Material and methods

### 2.1 Animal preparation

All animal procedures were approved and conducted in accordance with the regulations of the Hasanuddin University of Medicine Committee on animal research (approval no 672A). This study has been registered in Research Registry.com (with the registration number of researchregistry8381). 15 adult New Zealand white rabbits aged 6–9 months (weight range, 2.0–3.5 kg) were used as approved by the institution's committee for animal experimentation. The rabbits were kept in cages with food and drink tailored to their needs and were divided into three treatment groups with 5 individuals each.

### 2.2 Preparation of PRF membrane

Platelet rich fibrin is made from rabbit blood by obtaining 10 ml whole blood from the ear of the New Zealand white Rabbit without anticoagulant. The blood was kept in a tube and centrifuged (Duo Quattro Machine, Nice, France) at 3000 rpm for 10 min at 4 °C. The tube shows PRF is located in the middle layer, between the ‘cellless plasma’ at the top and the red blood cell layer below. Moreover, the PRF composed is in the form of a gel [19].

### 2.3 Surgical procedure

The rabbits were anesthetized by intramuscular injection of ketamine 50 mg/ml (Siegfried Hameln, Germany) and xylazine 0.2 mg/ml (Interchemie, Netherlands). Surgery was performed on the right knee, which was previously shaved, disinfected, and sterilely draped. The surgical procedure was carried out by an orthopedic surgeon. Also, the rabbit was given a preoperative injection of Cefazolin sodium an hour before the surgery (Cefazol, Dankos Farma, Indonesia). A medial parapatellar incision was used to approach and the patella was laterally dislocated. A full-thickness cartilage defect of 4-mm-diameter and 2.4-mm depth was marked in the trochlear groove of the femur with Kirschner-wire (Eka Ormed, Indonesia). Furthermore, microfracture was carried out by drilling with as many as 3 pieces of Kirschner-wire in the cartilage defect. The experimental animals were divided into 3 treatment groups. In group 1, only cartilage defects were made on the rabbit knees. In group 2, the defects were followed by microfracture. Meanwhile, in group 3, microfracture and synovium-platelet rich fibrin (S-PRF) grafting were performed. PRF was applied over the defect in such a way that it was covered and closed again with a synovial membrane. Subsequently, the synovium membrane was removed from the surrounding cartilage and attached with fibrin glue (Brand BioGlue, CryoLife, United States) to the surrounding cartilage. The cartilage defect is covered by two layers, namely the PRF and the synovium.

After completing the procedure, the patella was returned to its original position and properly sutured. The antibiotic cefazolin sodium was given for approximately 24 h after surgery at a dose of 75 mg/kg. After 12 weeks, the experimental animals were sacrificed by means of intravenous pentobarbital.

### 2.4 Macroscopic and histologic examination

The condyle, which has the cartilage defect was dissected and microscopic examination was immediately carried out. The cartilage healing tissue was macroscopically and microscopically assessed by the International Cartilage Repair Society (ICRS) as shown in Table 1 [20]. For macroscopic and histological examination, the score was assessed by 2 investigators and the final result was the average of these scores. One of the investigators is a surgeon and the other is an independent individual blinded to the treatment. Additionally, the histologic evaluation was conducted in a blinded manner.

**Table 1:** ICRS macroscopic evaluation of cartilage repair.

Categories	Score
Degree pf defect repair	
In level with surrounding cartilage	4
75% repair of defect depth	3
50% repair of defect depth	2
25% repair of defect depth	1
0% repair of defect depth	0
Integration to border zone	
Complete integration with surrounding cartilage	4
Demarcating border <1 mm	3
¾ of graft integrated, ¼ with a notable border >1 mm width	2
½ of graft integrated with surrounding cartilage, ½ with a notable border >1 mm	1
From no contact to ¼ of graft integrated with surrounding cartilage	0
Macroscopic appearance	
Intact smooth surface	4
Figrillated surface	3
Small, scattered fissures or cracks	2
Several, small or few but large fissures	1
Total degeneration of grafted area	0
Overall repair assessment	
Grade I: normal	12
Grade II: nearly normal	11–8
Grade III: abnormal	7–4
Grade IV: severely abnormal	3–1

For histological examination, the condyles were fixed with formalin, decalcified in nitric acid, and embedded in paraffin. Ten-micrometer-thick sagittal cross sections were cut through the tissue and stained with hematoxylin-eosin. The histologic result was evaluated with the International Cartilage Repair Society (ICRS) for a histological grading system as shown in Table 2 [21].

**Table 2:** ICRS visual histological assessment scale.

Feature	Score
I. Surface	
Smooth/continuous	3
Discontinulities/irregularities	0
II. Matrix	
Hyaline	3
Mixture: Hyaline/fibrocartilage	2
Fibrocartilage	1
Fibrous tissue	0
III. Cell distribution	
Columnar	3
Mixed/columnar-clusters	2
Clusters	1
IV. Cell population viability	
Predominantly viable	3
Partially viable	1
<10% viable	0
V. Subchondral Bone	
Norma	3
Increased remodeling	2
Bone necrosis/granulation tissue	1
Detached/fracture/callus at base	0
VI. Cartilage mineralization (calcified cartilage)	
Normal	3
Abnormal/inappropriate location	0

### 2.5 mRNA aggrecan and mRNA type 2 collagen expresion

Total RNA was extracted from the regenerated tissues in the defect area. A 100 g of fresh tissue was added to 900 μl of “L6” solution consisting 120 g of Guanidium thyocianate (GuSCN) (Fluka Chemie AG, Buchs, Switzerland, cat no. 50990) in 100 ml of 0.1 M Tris HCl, pH 6.4, 22 ml 0.2 M Ethylene Diamine Tetra Acetate (EDTA) pH 8.0 and 2.6 g Triton X-100 (Packard, Instruments) with a final concentration of 50 mM Tris HCl, 5 M GuSCN, 20 mM EDTA, and 0.1% Triton X-100. Subsequently, the mixture was centrifuged at 12,000 rpm and the sediment was added to a 20 μl diatom suspension consisting of 50 ml H2O and 500 μl of 32% (w/v) “Celite” (“diatom”) (Jansen Chimica, Beerse, Belgium). Moreover, 20 μl of this diatom suspension could bind 10 μg of tissue RNA, it was vortexed and centrifuged in a 1.5 ml Eppendorf tube at 12,000 rpm for 15 min. The supernatant was removed and the sediment was washed by adding 1 ml of “L2” solution which consist of 120 g of GuSCN in 100 ml 0.1 M Tris HCl, pH 6.4. It was vortexed and centrifuged at 12,000 rpm for 15 min, the washing was repeated 2 times using an “L2” solution, and subsequently with 1 ml of 70% ethanol 2 times and 1 ml of acetone. The resulting mixture was heated in a water bath at 56 °C for 10 min and 60 μl of “TE” solution consisting of 1 mM EDTA was added to 10 mM Tris HCL pH 8.0. Furthermore, it was vortexed and centrifuged at 12,000 rpm for 30 s, and incubated in the oven for 10 min at 56 °C. It was vortexed and re-centrifuged for 30 s at 12,000 rpm, and the supernatant was obtained. The supernatant from this process produced nucleotide extraction results and was stored at −80 °C before performing PCR analysis [22].

The primary nucleotide sequence of rabbit mRNA aggrecan and collagen type 2 used is Primer mRNA aggrecan: 5′-ATCTACCGCTGTGAGGTGAT-3′ (forward) and 5′-CTCCTGGAAGGTGAACTTCT-3′ (reverse). The next is for collagen type 2 primers: 5′-AAGAGCGGTGACTACTGGAT-3′ (forward) and 5′-ACGCTGTTCTTGCAGTGGTA-3 (reverse). Meanwhile, the housekeeping used by Rabbit GAPDH with its primary nucleotide sequence is 5′-GTCAAGGCTGAGAACGGGAA-3 (forward) and 5′-GCTTCACCACCTTCTTGATG-3′ (reverse). PCR conditions are the initial stage of activation with a temperature of 95 °C for 30 s, followed by 40 cycles at a temperature of 95 °C for 30 s and 60 °C for 30 s. The next amplification process is in accordance with the Fachri et al. protocol where qRT PCR uses a sybrgreen qRT-PCR master mix kit in one step. This protocol is optimized for the CFX Connect System (USA) real-time PCR machine instrument. The protocol was adjusted with the instrument by changing the dye dilution according to the manual instructions and following the manufacturer's recommended instrument for the RT-PCR cycle program [22,23].

Passive reference dye was included in the reaction, and diluted at 1:500. The solutions containing dyes are kept away from light. Also, dilute 2× SYBR Green QRT-PCR master was mixed and stored in ice. Following the initial defrosting of the master mix, the unused portion was stored at 4 °C on record, avoiding repeated freeze-thaw cycles. The reagent mixture had a final volume of 25 μl and sample mRNA was extracted according to the protocol. Each sample was carried out in triplicate (three replications). 12.5 μl of 2× SYBR Green QRT-PCR master mix was added x μl of initial primer (optimized concentration) plus Nuclease – PCR free – H2 level x μl final primer (optimized concentration). Moreover, 0.375 μl reference dye solution from step 1 (optional) and 1.0 μl of RT/Rnase block enzyme mixture with 25 μl total reaction volume can be used. The reaction was mixed slowly, hence, no bubble was formed (not rotated). The mixture was subsequently distributed into test tubes by adding x μl of experimental RNA to each tube and was briefly centrifuged and placed in the instrument. The PCR program was ready to run using a Real-time PCR machine (CFX Connect system, Biorad Laboratories, Real-Time PCR 96 well 0.1 ml, USA) [22,23]. The value of each control sample was set at 1 and used to calculate the fold change of target genes.

### 2.6 Statistical analysis

The analysis of variance test was used to compare macroscopic and histologic scores between the three groups. Meanwhile, the Kruskal-Wallis test was used to compare aggrecan and collagen type 2 expression. P < 0.05 was considered statistically significant. A power analysis was carried out with the power 0.8; α value and standard deviation set at 0.05 and 2.1.

## 3 Results

A total of 15 white New Zealand rabbits were used and equally divided into three groups. Furthermore, evaluation of defect regeneration results was carried out 12 weeks after treatment. The average weight of the rabbits was 2994.67 ± 10.45 g, and the p-value was ≥0.05.

A total of 10 ml rabbit blood without anticoagulant was directly centrifuged at 3000 rpm for 10 min. PRF, which is clearer in color and viscous is located in the center of the centrifugation tube. The released PRF will separate into two layers, above and below (cloud color).

**Fig. 1:**
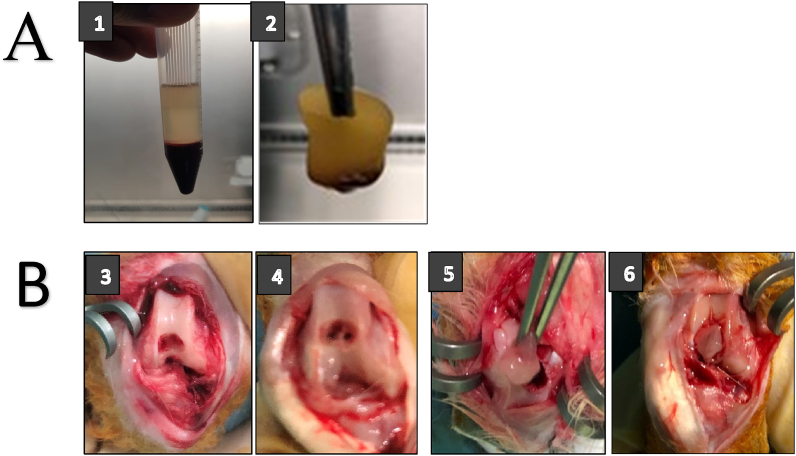
(A) The process of making PRF membrane. (1) PRF after centrifugation. (2) PRF separated. (B) The process of making cartilage defects, microfractures and transplantation of synovium-platelet rich fibrin (S-PRF). (3) Cartilage defect. (4) Cartilage defect and microfracture. (5) PRF Implantation. (6) Synovium Implantation over PRF.

The separated PRF is viscous and forms a membrane layer and this can be cut as needed (size) at the cartilage defect hole as indicated in Fig. 1A. These defects are created in the intercondylar depression of the rabbit femur. The defect was made at diameter of 3.5 mm and a depth of 2.4 mm as shown in Fig. 1B. In the defect, PRF was grafted in the form of a membrane and the synovium was transplanted. The cartilage hole was closed by two layers and glued with a fibrin glue (BioGlue) to the surrounding cartilage (Fig. 1B).

### 3.1 Macroscopic observation and ICRS score

**Fig. 2:**
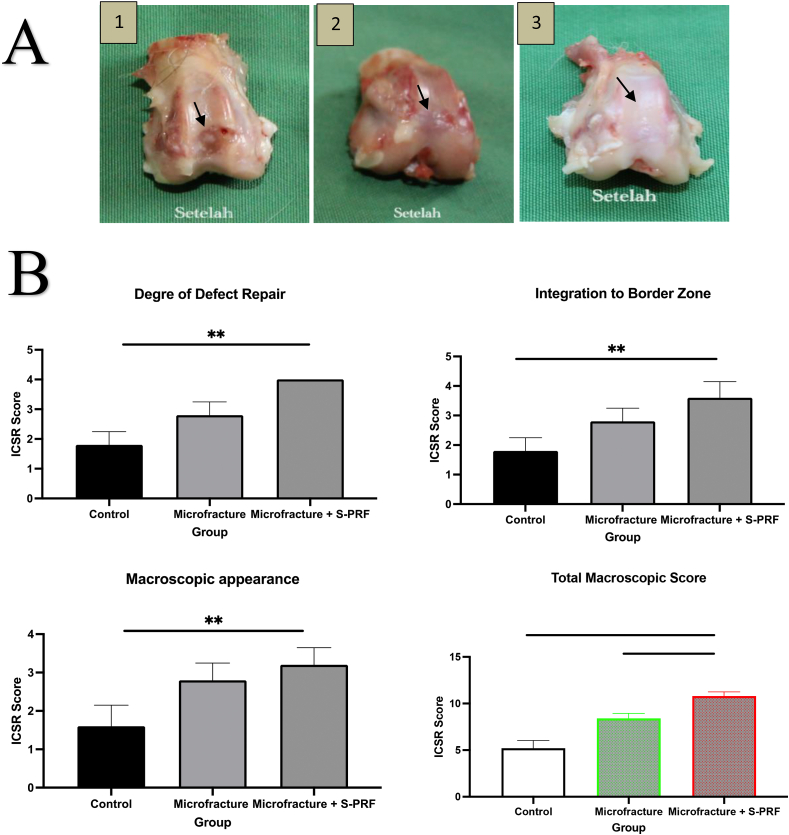
(A) Macroscopic results on cartilage healing organs (black arrows). (1) Control Group. (2) Microfracture group. (3) Microfracture + S-PRF group. (B) Macroscopic cartilage healed evaluation according to ICSR score: Degree of cartilage repair, Integration to border zone, Masroscopic appearance and total masroccopic score. *, P < 0.05; **, P < 0.01; and ***, P < 0.001. p < 0.05 was considered statistically significant.

On macroscopic examination indicated in Fig. 2A, it is shown that the knee cartilage of rabbits in the control group has not heal or completely filled. Although the cartilage was filled to the brim in the microfracture group, there were still hollows or scratches on the edges and the surface was uneven. Meanwhile, in the microfracture group + S-PRF, the cartilage was completely filled, and the surface was smooth and flat.

Macroscopic and histological evaluation was carried out 12 weeks after treatment. The evaluation used ICSR standards for macros and ICSRs for microscopic ones. The ICSR score on macroscopically consists of 4 categories, namely the degree of cartilage repair, integration with surrounding cartilage, macroscopic appearance, and overall repair assessment.

In the descriptive data, the mean score in the three categories and the total ICSR score was significantly higher in the microfracture treatment group and the microfracture + S-PRF treatment group compared to the control scores. In the macroscopic ICSR scores in the degree of healing category, integration with surrounding cartilage, and macroscopic appearance, the ICSR scores were found to be significantly higher Microfracture + S-PRF groups compared to the control but not significantly higher compared to microfracture group. The ICSR total score, the Microfracture + S-PRF group was significantly higher than the Microfracture (p < 0.00) and control (p < 0.000) as shown in Fig. 2B.

### 3.2 Histologic observation and ICRS score

**Fig. 3:**
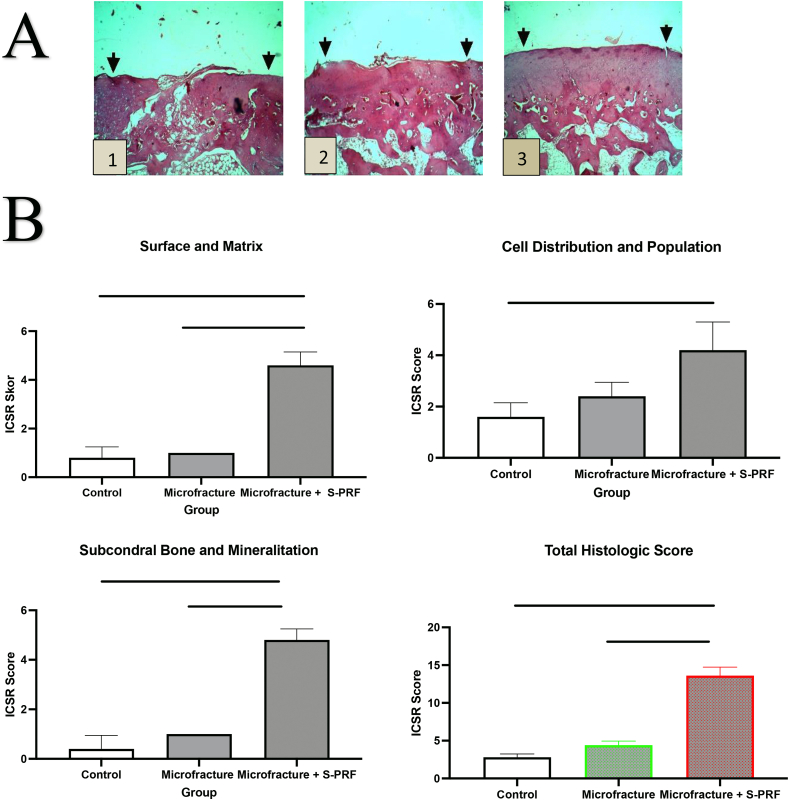
(A) Histological evaluation results on cartilage healing tissue. (1) Control group. (2) Microfracture group. (3) Microfracture + S-PRF. (B) Histologic cartilage healed evaluation according to ICSR score: surface and matrix, Cell distribution and population, subcondral bone and mineralitation and total histologic score. *, P < 0.05; **, P < 0.01; and ***, P < 0.001. p < 0.05 was considered statistically significant.

On histological examination (Fig. 3A), it is shown that the knee cartilage in the control group had an uneven surface, and the cartilage close to the subchondral layer had several cavities. Overall, the cartilage layer has not healed, but only partially filled with chondrocytes. In the microfracture group, the cartilage defects were mostly filled with chondrocytes but there were still chondroblasts (young chondrocyte cells), uneven surface, and several hypertrophic cells. Meanwhile, in the microfracture + S-PRF group, the cartilage was completely filled with chondrocytes, the surface was smooth, and the cartilage formed was integrated into the surrounding with indistinguishable boundaries.

Histological evaluation using ICSR standards for microscopy. ICSR scores on histological evaluation consists of 6 categories, namely the surface category, matrix, cell distribution, viable cell population, subchondral bone, and cartilage mineralization. In the descriptive data, the mean score of the three combined categories and the total ICSR score were significantly higher in the microfracture treatment group and the microfracture + S-PRF treatment group compared to the control group scores. In surface and matrix histology, subchondral and calcification categories, ICSR scores were significantly higher in the Microfracture + S-PRF group compared to the microfracture and control. However, the score of cell distribution and viability in the microfracture + S-PRF group was not different to microfracture group. In the total histological score, the scores in the Microfracture + S-PRF group were higher than the Microfracture (p < 0.00) and control (p < 0.000), as shown in Fig. 3B.

### 3.3 mRNA aggrecan and type 2 collagen expresion

The RT-PCR examination on healing of knee cartilage defects aims to determine the quantitative levels of mRNA expression of aggrecan genes and type 2 collagen.

**Fig. 4:**
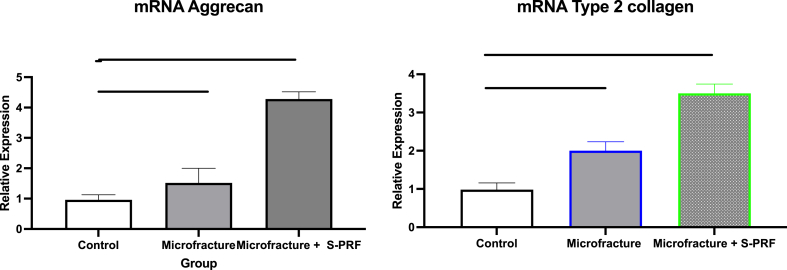
mRNA aggrecan and mRNA type 2 collagen expression on cartilage healed by RT-PCR examination. *, P < 0.05; **, P < 0.01; and p < 0.05 was considered statistically significant.

The mean relative expression of mRNA aggrecan and mRNA type 2 collagen on RT-PCR examination were the lowest in the control group. The levels were higher in the microfracture group and highest in the Microfracture + S-PRF group. mRNA Aggrecan expression levels on RT-PCR examination in the Microfracture + S-PRF group were found to be higher significantly than the microfracture group (p < 0.05) and the control (p < 0.01). Similarly, mRNA type 2 collagen relative expression in the Microfracture + S-PRF group were found to be higher significantly than the microfracture (p < 0,05) and the control groups (p < 0.01) as shown in Fig. 4.

## 4 Discussion

Microfracture is a procedure of drilling in damaged cartilage to aid the bone marrow stimulate a spontaneous repair reaction. The procedure allows MSCs and growth factors from the bone marrow to reach the cartilage defect. However, the bone marrow stimulation procedure with microfractures has limitations, particularly in the treatment of extensive chondral and osteochondral lesions where the outcome is difficult to predict [13,14]. The healing of cartilage defects with microfractures produces fibrocartilage which is mainly composed of type 1 collagen. Although the healing tissue is able to fill the defect, the composition and biomechanics are under normal histology. Therefore, this network is unstable to compression or shear forces and tends to degenerate over time [14,24].

PRF is a product made by centrifugation of blood obtained from the body without the addition of anticoagulants. PRF contains platelets, leukocytes, and several growth factors, such as platelet-derived GF (PDGF), insulin-like GF (IGF-1), transforming GF-β1 (TGF-β1), vascular endothelial GF (VEGF), basic fibroblastic GF (bFGF), and epidermal GF (EGF) [19,25] Furthermore, it contains a lot of cells such as stem cells trapped in the fibrin network which makes it more solid, therefore, besides having the potential to regenerate, it is also easier to apply [26].

The application of PRF in the field of final regeneration has expanded to the field of cartilage and tendon healing. Growth factor derivatives, such as PDGF, TGF-β1, and IGF-1 can act as stimulators for chondrogenesis, tenogenesis by regulating proliferation, inflammation, neo-angiogenesis, and extracellular matrix (ECM) deposition. Concurrent administration of growth factors in the form of administration of blood products as in PRF can overcome this deficiency [17,25]. Several studies have shown that growth factors as bioactive can improve the healing of cartilage injuries and reduce or slow down the degeneration of osteoarthritis. This growth factor is an anabolic factor for cartilage formation. They work by stimulating chondrocytes to synthesize proteoglycans, aggrecans, and type 2 collagen including stimulating proliferation, directing chondrogenic stem cell differentiation, and inhibiting the catabolic effects of cytokines [27].

The synovium is a thin tissue that lines the joint surface, in which the underlying layer contains a mixture of chondroprogenitor, macrophages, and fibroblast cells. Cells in the synovium have almost the same potential as mesenchymal stem cells. The cell source for tissue engineering techniques is synovium [18]. In vitro, these synovial cells are known to be chondrogenic and capable of differentiation into chondroblasts. Synovium contains cells that can differentiate into chondrocytes when given certain growth factors [28].

The tissue cover in the defect area helps to hold cells in the early stages of granulation tissue formation, therefore, preventing the release of mesenchymal and anabolic mediator cells from the repair site. As a tissue cover in this study, synovium has several advantages, such as being a natural tissue, easy to obtain and apply, as well as having chondrogenic properties. Furthermore, it can function to protect or stabilize blood clots in the defect and can also increase the chondrogenic differentiation of the mesenchymal cells [28]. The synovium has chondrogenic properties similar to those of modern tissues. The covering of the tissue with synovium can prevent subchondral bone thickening, subchondral cyst formation, and the presence of intralesional osteophytes, which are often found in patients treated with microfractures only [8]. Moreover, it can prevent implantation failure which often occurs in the use of autologous chondrocytes due to the synovium characteristics [3,29].

This study showed that the expression of aggrecan and type 2 collagen was higher in cartilage healing tissue that underwent microfracture and synovium - PRF transplantation. In a study related to the effect of microfracture treatment and administration of platelet-rich plasma on cartilage defects, there was an increase in the expression of type 2 collagen in healing tissues. This increase occurs due to increased cell activity, synthesis of extra-cellular material, increased cell migration, and stimulation of subchondral progenitor cells [30]. In in vitro studies, there was an increase in the number of cells due to the proliferation of progenitor cells. These cells move closer to form contact with others for the next stage of chondrogenesis, where chondroprogenitor cells express type 2 collagen as well as aggrecans and simultaneously downregulate type 1 collagen [31].

In another study where cartilage defects were treated with a combination of microfractures and the administration of PRF membrane in one stage of action, the results of cartilage healing were better macroscopically and microscopically [20]. Similarly, a study which combined microfractures and PRF showed that the administration of PRF increased the repair of cartilage defects macroscopically and histologically. PRF as a source of growth factors enhances this cartilage repair [16,32]. This is because the mechanism for healing the defects, especially at the stage of cell migration, is activated by growth factors [33]. The use of PRP could increase healing in musculoskeletal injury and, in experimental animals, can also increase the integration of the osteochondral graft with the surrounding cartilage tissue and inhibit degeneration [30,34]. PRF can maintain hyaline cartilage on osteochondral autograft more than PRP, and has the potential to enhance clinical outcomes of osteochondral autograft or cartilage transplantation used to treat osteochondral lesions [35,36].

Growth factors present in platelet-rich fibrin can direct mesenchymal stem cells from the bone marrow and synovium to differentiate or proliferate into chondroblasts. Subsequently, chondroblasts become chondrocytes and form a cartilage matrix, including aggrecan and type 2 collagen [28]. MSCs from bone marrow in synovium and growth factors in PRF should be able to grow optimally into hyaline-like cartilage since the cells can be maintained, attached, and fused at the site of cartilage defects. One of the factors that made the microfracture results unsatisfactory is the grafted cells, which did not survive at the site of the cartilage defect. The dynamic nature of the joint synovial fluid is the same as that of joints that are always moving, causing chondrocytes or MSCs difficult to be attached to the injured cartilage area [29,37]. Other factors thought to be the cause are the few number of MSCs that go to the injury site and the extremely low number of growth factors. It is also suspected that the progenitor cells from the bone marrow are more likely to spread to the joint fluid, therefore, only few are attached to the injury site [16,37].

However, this weakness can be overcome by the synovium-PRF covered microfracture method. Cartilage defects are covered with two membrane layers, namely synovium and PRF where the former is rich in mesenchymal cells and the latter in growth factors. In this study, besides PRF being a source of growth factors, it also acts as a scaffold for cartilage healing [16]. Platelet-rich fibrin is in the form of a membrane, hence it is easy to apply to the surface of deformed cartilage. Meanwhile, synovium itself is a cell-rich network in the form of a membrane that is easily applied over the PRF. The PRF membranes has previously been widely used in dentistry and promising results were obtained [17].

The disadvantages of using ACI and MSC tissue engineering technology are the technology and facilities needed to develop stem cells which are quite complex, the extensive time required to prepare the cells, double operation is needed, and the large costs [8]. Currently, there is a tendency for cartilage defect healing procedures to lead to simple processes, such as eliminating the procedure twice, utilizing natural scaffolds derived from the patient's own body with a simple process and no need to suture the process of closing the defect, as well as eliminating procedures that require cell culture [38]. This poses a challenge for cartilage treatment in developing countries with limited equipment, technology, and costs. PRF has the potential to increase chondrocyte migration, viability, cellular proliferation and differentiation. This benefit fully improves cartilage repair, attainable at 1 stage, culture-free method of combining PRF and autologous cartilage graft to repair joint chondral defects [39].

The surgical procedure for healing cartilage defects with microfractures augmented by PRF and synovium transplantation could be the solution to this problem. This procedure has several advantages as follows, first, the procedure for making PRF is simpler, faster, less expensive, and does not require complicated preparation of actions. Second, this surgical procedure requires only one step and does not need material collection or prior cell culture. Third, it does not require an additional external scaffold because the synovium-PRF can function as a membrane-shaped scaffold. Scaffold produced from outside (synthetic) costs money, has inflammation risk, and the procedure is more complicated.

### 4.1 Limitations

The limitations of this study are as follows, first, the healing between rabbits and humans is different, as that of the animals is better. Second, there was no mechanical evaluation of the cartilage healing tissue. Third, there could be subjective bias in the result evaluation, either macroscopically or microscopically. Fourth, it is still unclear which part is more dominant in healing, either the synovium, bone marrow cells, growth factors, or the combined action of platelet-rich fibrin, synovium, and bone marrow.

## 5 Conclusions

This study showed that microfractures and transplantation of synovium-platelet rich fibrin (S-PRF) can regenerate knee cartilage defects. This is proved by the increased expression of aggrecan mRNA and type 2 collagen mRNA in cartilage healing, as well as macroscopic and histological evaluation which yielded the best improvement.

## Author contribution statement

Ahmad Taufik S: Conceived and designed the experiments; Performed the experiments; Analyzed and interpreted the data; Contributed reagents, materials, analysis tools or data; Wrote the paper.

Bayu Tirta Dirja, Dwikora Novembri Utomo, Muhamad Andry Usman: Conceived and designed the experiments; Performed the experiments; Analyzed and interpreted the data; Wrote the paper.

Muhammad Sakti, Muhammad Ruksal Saleh: Conceived and designed the experiments; Performed the experiments; Analyzed and interpreted the data.

Mochammad Hatta: Conceived and designed the experiments; Contributed reagents, materials, analysis tools or data; Wrote the paper.

Budu: Conceived and designed the experiments; Performed the experiments; Analyzed and interpreted the data; Contributed reagents, materials, analysis tools or data.

## Funding statement

This research did not receive any specific grant from funding agencies in the public, commercial, or not-for-profit sectors.

## Data availability statement

Data included in article/supp. material/referenced in article.

## Additional information

This study has been reviewed and approved by Hasanuddin University of Medicine Committee on animal research (approval no 672A).

We have registered our study in “Research Registry” with registration number 0f “researchregistry8381”.

## Declaration of interest's statement

The authors declare no competing interests.
